# Comparative effectiveness research for the clinician researcher: a framework for making a methodological design choice

**DOI:** 10.1186/s13063-016-1535-6

**Published:** 2016-08-17

**Authors:** Cylie M. Williams, Elizabeth H. Skinner, Alicia M. James, Jill L. Cook, Steven M. McPhail, Terry P. Haines

**Affiliations:** 1 Peninsula Health, Community Health, PO Box 52, Frankston, Melbourne, Victoria 3199 Australia; 2 Monash University, School of Physiotherapy, Melbourne, Australia; 3 Monash Health, Allied Health Research Unit, Melbourne, Australia; 4 Western Health, Allied Health, Melbourne, Australia; 5 Queensland University of Technology, School of Public Health and Social Work, Brisbane, Australia

**Keywords:** dissent and dispute, health economics, hospital, patient safety, research design

## Abstract

Comparative effectiveness research compares two active forms of treatment or usual care in comparison with usual care with an additional intervention element. These types of study are commonly conducted following a placebo or no active treatment trial. Research designs with a placebo or non-active treatment arm can be challenging for the clinician researcher when conducted within the healthcare environment with patients attending for treatment.

A framework for conducting comparative effectiveness research is needed, particularly for interventions for which there are no strong regulatory requirements that must be met prior to their introduction into usual care. We argue for a broader use of comparative effectiveness research to achieve translatable real-world clinical research. These types of research design also affect the rapid uptake of evidence-based clinical practice within the healthcare setting.

This framework includes questions to guide the clinician researcher into the most appropriate trial design to measure treatment effect. These questions include consideration given to current treatment provision during usual care, known treatment effectiveness, side effects of treatments, economic impact, and the setting in which the research is being undertaken.

## Background

Comparative effectiveness research compares two active forms of treatment or usual care in comparison with usual care with an additional intervention element. Comparative effectiveness research differs from study designs that have an inactive control, such as a ‘no-intervention’ or placebo group. In pharmaceutical research, trial designs in which placebo drugs are tested against the trial medication are often labeled ‘Phase III’ trials. Phase III trials aim to produce high-quality evidence of intervention efficacy and are important to identify potential side effects and benefits. Health outcome research with this study design involves the placebo being non-treatment or a ‘sham’ treatment option [1].

Traditionally, comparative effectiveness research is conducted following completion of a Phase III placebo control trial [2–4]. It is possible that comparative effectiveness research might not determine whether one treatment has clinical beneficence, because the comparator treatment might be harmful, irrelevant, or ineffective. This is unless the comparator treatment has already demonstrated superiority to a placebo [2]. Moreover, comparing an active treatment to an inactive control will be more likely to produce larger effect sizes than a comparison of two active treatments [5], requiring smaller sample sizes and lower costs to establish or refute the effectiveness of a treatment. Historically, then, treatments only become candidates for comparative effectiveness research to establish superiority, after a treatment has demonstrated efficacy against an inactive control.

Frequently, the provision of health interventions precedes development of the evidence base directly supporting their use [6]. Some service-provision contexts are highly regulated and high standards of evidence are required before an intervention can be provided (such as pharmacological interventions and device use). However, this is not universally the case for all services that may be provided in healthcare interventions. Despite this, there may be expectation from the individual patient and the public that individuals who present to a health service will receive some form of care deemed appropriate by treating clinicians, even in the absence of research-based evidence supporting this. This expectation may be amplified in publicly subsidized health services (as is largely the case in Canada, the UK, Australia, and many other developed nations) [7–9]. If a treatment is already widely employed by health professionals and is accepted by patients as a component of usual care, then it is important to consider the ethics and practicality of attempting a placebo or no-intervention control trial in this context. In this context, comparative effectiveness research could provide valuable insights to treatment effectiveness, disease pathophysiology, and economic efficiency in service delivery, with greater research feasibility than the traditional paradigm just described. Further, some authors have argued that studies with inactive control groups are used when comparative effectiveness research designs are more appropriate [10]. We propose and justify a framework for conducting research that argues for the broader use of comparative effectiveness research to achieve more feasible and translatable real-world clinical research.

This debate is important for the research community; particularly those engaged in the planning and execution of research in clinical practice settings, particularly in the provision of non-pharmacological, non-device type interventions. The ethical, preferential, and pragmatic implications from active versus inactive comparator selection in clinical trials not only influence the range of theoretical conclusions that could be drawn from a study, but also the lived experiences of patients and their treating clinical teams. The comparator selection will also have important implications for policy and practice when considering potential translation into clinical settings. It is these implications that affect the clinical researcher’s methodological design choice and justification.

## Framework

**Fig. 1:**
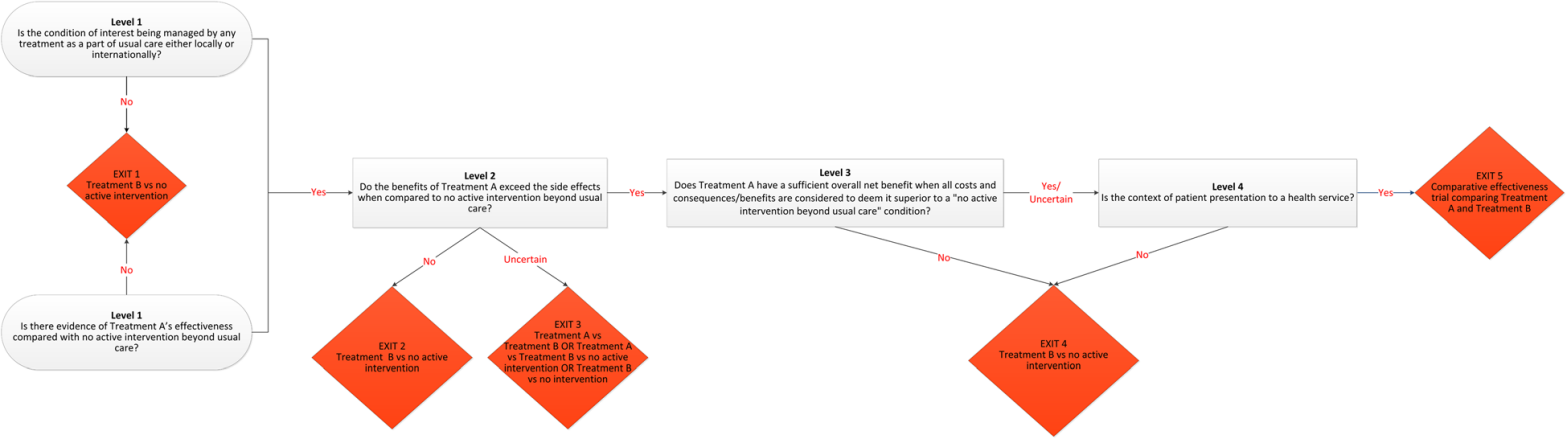
Comparative effectiveness research decision-making framework. Treatment A represents any treatment for a particular condition, which may or may not be a component of usual care to manage that condition. Treatment B is used to represent our treatment of interest. Where the response is unknown, the user should choose the NO response

The decision-making framework takes the form of a decision tree (Fig. 1) to determine when a comparative effectiveness study can be justified and is particularly relevant to the provision of services that do not have a tight regulatory framework governing when an intervention can be used as part of usual care. This framework is headed by Level 1 questions (demarcated by a question within an oval), which feed into decision nodes (demarcated by rectangles), which end in decision points (demarcated by diamonds). Each question is discussed with clinical examples to illustrate relevant points.

Treatment A is any treatment for a particular condition that may or may not be a component of usual care to manage that condition. Treatment B is our treatment of interest. The framework results in three possible recommendations: that either (i) a study design comparing Treatment B with no active intervention could be used, or (ii) a study design comparing Treatment A, Treatment B and no active intervention should be used, or (iii) a comparative effectiveness study (Treatment A versus Treatment B) should be used.

## Level 1 questions

### Is the condition of interest being managed by any treatment as part of usual care either locally or internationally?

Researchers first need to identify what treatments are being offered as usual care to their target patient population to consider whether to perform a comparative effectiveness research (Treatment A versus B) or use a design comparing Treatment B with an inactive control. Usual care has been shown to vary across healthcare settings for many interventions [11, 12]; thus, researchers should understand that usual care in their context might not be usual care universally. Consequently, researchers must consider what comprises usual care both in their local context and more broadly.

If there is no usual care treatment, then it is practical to undertake a design comparing Treatment B with no active treatment (Fig. 1, Exit 1). If there is strong evidence of treatment effectiveness, safety, and cost-effectiveness of Treatment A that is not a component of usual care locally, this treatment should be considered for inclusion in the study. This situation can occur from delayed translation of research evidence into practice, with an estimated 17 years to implement only 14 % of research in evidence-based care [13]. In this circumstance, although it may be more feasible to use a Treatment B versus no active treatment design, the value of this research will be very limited, compared with comparative effectiveness research of Treatment A versus B. If the condition is currently being treated as part of usual care, then the researcher should consider the alternate Level 1 question for progression to Level 2.

As an example, prevention of falls is a safety priority within all healthcare sectors and most healthcare services have mitigation strategies in place. Evaluation of the effectiveness of different fall-prevention strategies within the hospital setting would most commonly require a comparative design [14]. A non-active treatment in this instance would mean withdrawal of a service that might be perceived as essential, a governmental health priority, and already integrated in the healthcare system.

### Is there evidence of Treatment A’s effectiveness compared with no active intervention beyond usual care?

If there is evidence of Treatment A’s effectiveness compared with a placebo or no active treatment, then we progress to Question 3. If Treatment A has limited evidence, a comparative effectiveness research design of Treatment B versus no active treatment design can be considered. By comparing Treatment A with Treatment B, researchers would generate relevant research evidence for their local healthcare setting (is Treatment B superior to usual care or Treatment A?) and other healthcare settings that use Treatment A as their usual care. This design may be particularly useful when the local population is targeted and extrapolation of research findings is less relevant.

For example, the success of chronic disease management programs (Treatment A) run in different Aboriginal communities were highly influenced by unique characteristics and local cultures and traditions [15]. Therefore, taking Treatment A to an urban setting or non-indigenous setting with those unique characteristics will render Treatment A ineffectual. The use of Treatment A may also be particularly useful in circumstances where the condition of interest has an uncertain etiology and the competing treatments under consideration address different pathophysiological pathways. However, if Treatment A has limited use beyond the research location and there are no compelling reasons to extrapolate findings more broadly applicable, then Treatment B versus no active control design may be suitable.

The key points clinical researchers should consider are:

- The commonality of the treatment within usual care
- The success of established treatments in localized or unique population groups only
- Established effectiveness of treatments compared with placebo or no active treatment

## Level 2 questions

### Do the benefits of Treatment A exceed the side effects when compared with no active intervention beyond usual care?

Where Treatment A is known to be effective, yet produces side effects, the severity, risk of occurrence, and duration of the side effects should be considered before it is used as a comparator for Treatment B. If the risk or potential severity of Treatment A is unacceptably high or is uncertain, and there are no other potential comparative treatments available, a study design comparing Treatment B with no active intervention should be used (Fig. 1, Exit 2). Whether Treatment A remains a component of usual care should also be considered. If the side effects of Treatment A are considered acceptable, comparative effectiveness research may still be warranted.

The clinician researcher may also be challenged when the risk of the Treatment A and risk of Treatment B are unknown or when one is marginally more risky than the other [16]. Unknown risk comparison between the two treatments when using this framework should be considered as uncertain and the design of Treatment A versus Treatment B or Treatment B versus no intervention or a three-arm trial investigating Treatment A, B and no intervention is potentially justified (Fig. 1, Exit 3).

A good example of risk comparison is the use of exercise programs. Walking has many health benefits, particularly for older adults, and has also demonstrated benefits in reducing falls [17]. Exercise programs inclusive of walking training have been shown to prevent falls but brisk walking programs for people at high risk of falls can increase the number of falls experienced [18]. The pragmatic approach of risk and design of comparative effectiveness research could better demonstrate the effect than a placebo (no active treatment) based trial.

The key points clinical researchers should consider are:

- Risk of treatment side effects (including death) in the design
- Acceptable levels of risk are present for all treatments

## Level 3 question

### Does Treatment A have a sufficient overall net benefit, when all costs and consequences or benefits are considered to deem it superior to a ‘no active intervention beyond usual care’ condition?

Simply being effective and free of unacceptable side effects is insufficient to warrant Treatment A being the standard for comparison. If the cost of providing Treatment A is so high that it renders its benefits insignificant compared with its costs, or Treatment A has been shown not to be cost-effective, or the cost-effectiveness is below acceptable thresholds, it is clear that Treatment A is not a realistic comparator. Some have advocated for a cost-effectiveness (cost-utility) threshold of $50,000 per quality-adjusted life year gained as being an appropriate threshold, though there is some disagreement about this and different societies might have different capacities to afford such a threshold [19]. Based on these considerations, one should further contemplate whether Treatment A should remain a component of usual care. If no other potential comparative treatments are available, a study design comparing Treatment B with no active intervention is recommended (Fig. 1, Exit 4).

If Treatment A does have demonstrated efficacy, safety, and cost-effectiveness compared with no active treatment, it is unethical to pursue a study design comparing Treatment B with no active intervention, where patients providing consent are being asked to forego a safe and effective treatment that they otherwise would have received. This is an unethical approach and also unfeasible, as the recruitment rates could be very poor. However, Treatment A may be reasonable to include as a comparison if it is usually purchased by the potential participant and is made available through the trial.

The methodological design of a diabetic foot wound study illustrates the importance of health economics [20]. This study compared the outcomes of Treatment A (non-surgical sharps debridement) with Treatment B (low-frequency ultrasonic debridement). Empirical evidence supports the need for wound care and non-intervention would place the patient at risk of further wound deterioration, potentially resulting in loss of limb loss or death [21]. High consumable expenses and increased short-term time demands compared with low expense and longer term decreased time demands must also be considered. The value of information should also be considered, with the existing levels of evidence weighed up against the opportunity cost of using research funds for another purpose in the context of the probability that Treatment A is cost-effective [22].

The key points clinical researchers should consider are:

- Economic evaluation and effect on treatment
- Understanding the health economics of treatment based on effectiveness will guide clinical practice
- Not all treatment costs are known but establishing these can guide evidence-based practice or research design

## Level 4 question

### Is the patient (potential participant) presenting to a health service or to a university- or research-administered clinic?

If Treatment A is not a component of usual care, one of three alternatives is being considered by the researcher: (i) conducting a comparative effectiveness study of Treatment B in addition to usual care versus usual care alone, (ii) introducing Treatment A to usual care for the purpose of the trial and then comparing it with Treatment B in addition to usual care, (iii) conducting a trial of Treatment B versus no active control. If the researcher is considering option (i), usual care should itself be considered to be Treatment A, and the researcher should return to Question 2 in our framework.

There is a recent focus on the importance of health research conducted by clinicians within health service settings as distinct from health research conducted by university-based academics within university settings [23, 24]. People who present to health services expect to receive treatment for their complaint, unlike a person responding to a research trial advertisement, where it is clearly stated that participants might not receive active treatment. It is in these circumstances that option (ii) is most appropriate.

Using research designs (option iii) comparing Treatment B with no active control within a health service setting poses challenges to clinical staff caring for patients, as they need to consider the ethics of enrolling patients into a study who might not receive an active treatment (Fig. 1, Exit 4). This is not to imply that the use of a non-active control is unethical. Where there is no evidence of effectiveness, this should be considered within the study design and in relation to the other framework questions about the risk and use of the treatment within usual care. Clinicians will need to establish the effectiveness, safety, and cost-effectiveness of the treatments and their impact on other health services, weighed against their concern for the patient’s well-being and the possibility that no treatment will be provided [25]. This is referred to as clinical equipoise.

Patients have a right to access publicly available health interventions, regardless of the presence of a trial. Comparing Treatment B with no active control is inappropriate, owing to usual care being withheld. However, if there is insufficient evidence that usual care is effective, or sufficient evidence that adverse events are likely, the treatment is prohibitive to implement within clinical practice, or the cost of the intervention is significant, a sham or placebo-based trial should be implemented.

Comparative effectiveness research evaluating different treatment options of heel pain within a community health service [26] highlighted the importance of the research setting. Children with heel pain who attended the health service for treatment were recruited for this study. Children and parents were asked on enrollment if they would participate if there were a potential assignment to a ‘no-intervention’ group. Of the 124 participants, only 7 % (*n* = 9) agreed that they would participate if placed into a group with no treatment [26].

The key points clinical researchers should consider are:

- The research setting can impact the design of research
- Clinical equipoise challenges clinicians during recruitment into research in the healthcare setting
- Patients enter a healthcare service for treatment; entering a clinical trial is not the presentation motive

## Conclusion

This framework describes and examines a decision structure for comparator selection in comparative effectiveness research based on current interventions, risk, and setting. While scientific rigor is critical, researchers in clinical contexts have additional considerations related to existing practice, patient safety, and outcomes. It is proposed that when trials are conducted in healthcare settings, a comparative effectiveness research design should be the preferred methodology to placebo-based trial design, provided that evidence for treatment options, risk, and setting have all been carefully considered.
